# BCR-ABL mutation testing to predict response to tyrosine kinase inhibitors in patients with chronic myeloid leukemia

**DOI:** 10.1371/currents.RRN1204

**Published:** 2011-01-11

**Authors:** Teruhiko Terasawa, Issa Dahabreh, Thomas A Trikalinos

**Affiliations:** ^*^Department of Internal Medicine, Fujita Health University School of Medicine; ^†^ICRHPS, Tufts Medical Center and ^‡^Tufts Evidence-based Practice Center, Institute for Clinical Research and Health Policy Studies, Tufts Medical Center

## Abstract

Tyrosine kinase inhibitors (TKIs) have revolutionized the treatment of chronic myeloid leukemia (CML). Although randomized evidence demonstrates that imatinib (a commercially available TKI) prolongs event–free survival in patients with CML, some patients develop imatinib intolerance or resistance. In addition, imatinib is less effective in patients who have progressed to more advanced disease stages, such as accelerated phase and blastic phase CML. For these reasons, 2nd generation TKIs that can inhibit the BCR-ABL protein more effectively or target additional disease mechanisms have been developed. Two such drugs have also been approved for clinical use by the FDA, nilotinib and dasatinib. Resistance to TKI treatment is thought to be mediated through various mechanisms, the most common of which is BCR-ABL1 mutations. Testing for mutations in BCR-ABL1 may predict lack of response to imatinib or may inform the choice between alternative TKIs.

## **Clinical Scenario**

           Chronic myeloid or myelogenous leukemia (CML) is a relatively uncommon hematological malignancy with approximately 5,000 new cases diagnosed annually in the USA [1]. Tyrosine kinase inhibitors (TKIs), small molecule drugs that target the ATP binding domain of tyrosine kinase enzymes, have revolutionized the treatment of CML[2] [3] [4]. Although randomized evidence demonstrates that imatinib (a commercially available TKI, currently approved by the Food and Drug Administration, FDA, for newly diagnosed CML in any disease stage) prolongs event–free and overall survival in patients with CML, some patients develop imatinib intolerance or resistance [5]. In addition, imatinib is less effective in patients who have progressed to more advanced disease stages, such as accelerated phase (AP) and blastic phase (BP) CML. For these reasons, 2nd generation TKIs that can inhibit the BCR-ABL protein more effectively or target additional disease mechanisms have been developed [6]. Two such drugs have also been approved for clinical use by the FDA, nilotinib and dasatinib [7] [8].     Resistance to TKI treatment is thought to be mediated through various mechanisms, including BCR-ABL1 mutations affecting the ATP binding domain, BCR-ABL1 amplification and overexpression, the acquisition of additional cytogenetic abnormalities by the leukemic clone, and expression of drug resistance proteins. BCR-ABL1 mutations are believed to represent the most common resistance mechanism and are more common in individuals with AP or BP CML [9]. Mutations can affect different amino acid residues of the BCR-ABL1 chimeric protein; more than 70 mutations have been reported to date and, based on clinical and in vitro data, they appear to confer differential sensitivity to TKIs.      Testing for mutations in the BCR-ABL gene may predict lack of response to imatinib or may inform the choice between alternative TKIs, which could be useful for clinical decision-making.

Three testing scenarios are common in clinical studies of *BCR-ABL1* mutations: 

 ·         Testing of patients with CML, both at an early stage (chronic phase [CP]) or with more advanced disease (AP and BP), to predict imatinib resistance. 

 ·         Testing of patients with CML who have imatinib resistance or intolerance to predict resistance to 2^nd^ generation inhibitors. 

 ·         Assessing of mutational status under treatment with TKIs for early detection of resistance development (monitoring of mutational status).

##  **Test Description**

    Analysis of multiple acquired somatic mutations in the chimeric BCR-ABL1 gene by complementary DNA based methods. Several methods have been developed for mutation detection, including direct sequencing, allele specific oligonucleotide polymerase chain reaction (ASO-PCR), matrix-assisted laser desorption/ionization time-of-flight (MALDI-TOF) spectrometry, pyrosequencing, high-resolution melting (HRM) curve analysis, and denaturing high performance liquid Chromatography (DHPLC).  

## **Public Health Importance**

    Before the use of TKIs became widespread, CML treatment was limited to stem cell transplantation (for eligible individuals) or combinations of drugs with relatively limited efficacy, such as hydroxyurea, interferon-alpha and cytarabine. In the pre-TKI era CML was associated with high mortality: based on a data from the Surveillance, Epidemiology, and End Results (SEER) Program of the National Cancer Institute, only 13% of patients diagnosed with CML were alive 5 years post-diagnosis [10]. The International Randomized Study of Interferon and STI571 (IRIS) demonstrated that imatinib results in improved survival free from progression to AP or BP and increased response rate, compared to a combination of interferon-alpha and cytarabine [2] [11]; however the benefits of imatinib therapy on survival may be less pronounced outside the clinical trial setting [12]. Studies based on the SEER database demonstrated that CML-related mortality declined during the period when imatinib became widely available [13]. Because of the increase in overall survival of patients with CML, the prevalence of the disease is expected to rise. In addition, as the number of individuals receiving imatinib treatment increases, the numbers of patients who require disease monitoring and those who develop resistance necessitating treatment with 2^nd^ generation TKIs are also expected to increase.

      Both in North and South America and in Europe there is documented variability in the implementation of mutational testing [14] [15]. A 2007 survey of USA and Europe-based physicians who treated at least 4 individuals with CML demonstrated that 60% of USA respondents were either unfamiliar with mutational testing or had never ordered the test.  

      For patients with newly diagnosed disease the choice between therapeutic strategies may be guided by mutational assessment at baseline. For patients developing resistance, treatment with increased in imatinib doses, switching to a second generation TKI or high-dose chemotherapy with stem cell transplantation are viable strategies and mutational testing may be used to select between them. Finally, in vitro and clinical differences in the sensitivity of the various mutations to 2^nd^ generation TKIs may be used to guide TKI choice. 

## **Published Reviews, Recommendations and Guidelines** 

**Systematic evidence reviews**

     Agency for Healthcare Research and Quality (AHRQ), Evidence Report/Technology Assessment [16].  

**Recommendations by independent group**

    The national Comprehensive Cancer Network guidelines for CML provide guidance for BCR-ABL1 mutational testing and specific algorithms for clinical implementation [17].

    The European Leukemia Network recently updated their recommendations for the management and monitoring of CML, including guidance regarding BCR-ABL1 mutations [18] [19].

## **Evidence Overview** 

***Analytic Validity*:**Test accuracy and reliability in measuring *[indicate analytes or other entities measured]* (analytic sensitivity and specificity).

    Test sensitivity for identifying *BCR-ABL1* mutations differs between the available methods. Direct sequencing has a detection sensitivity of 10-20% and can detect all mutations present in the sequenced region. DHPLC, double gradient denaturing electrophoresis, pyrosequencing, HRM curve analysis and array-based analyses have a sensitivity of 1-5%. Nucleic acid-based PCR clamping and ASO-PCR have even higher sensitivity but can only detect specific (known) mutations. A comparative study, concluded that high sensitivity methods detect a large number of low-level mutations of unknown clinical significance [20]. 

      The frequency of failed tests is unclear.

***Clinical Validity*:**Test accuracy and reliability in *[supporting clinical or public health assessment]* (predictive value).

    Test accuracy and reliability in predicting clinical outcomes such as response to treatment. Based on an AHRQ Draft Technology Assessment that included 31 studies [16].

 ·       The majority of evidence pertains to the short term surrogate outcomes of hematologic, cytogenetic or molecular response. Data on overall or progression-free survival are sparse.

 ·       The presence of *any* *BCR-ABL1* mutation (that is when considering all mutations together) does not appear to predict differential response to tyrosine kinase inhibitor (TKI) treatments (defined as imatinib-, dasatinib-, and nilotinib-based regimens). 

 ·       There is consistent evidence that presence of the relatively rare T315I mutation can predict TKI treatment failure, mainly in terms of hematologic and cytogenetic response. 

 ·       The fact that presence of *any BCR-ABL1 *mutation does not appear to differentiate response to TKI therapies is emblematic of the complexity of this topic: different mutations may confer different resistance to each of the three drugs. Exploring such relationships with systematic reviews of published aggregate data is extremely challenging. Other approaches, including collaborative registries of CML patients are much better suited to address such questions.

 ·       Most evidence is on second line TKI treatments, especially dasatinib and nilotinib, and originates from a small number of referral cancer centers where those agents were first-tested before becoming more widely available.

 ·       Evidence on pre- or early-therapy mutation testing or periodic monitoring of mutational status during treatment to predict resistance to a TKI is limited.

Recent additions to the literature include:

 ·      The European Leukemia Net updated management recommendations for CML [19]. The recommendations are based on expert consensus and non-systematic literature searches and provide guidance on implementing mutational testing. 

 ·       Two randomized controlled trials comparing imatinib to the use of second generation TKIs for the treatment of newly diagnosed CML [21] [22].

 ·       Recent expert guidance on the use of mutational testing [23] [24].

*****Clinical Utility*: **Net benefit of test in improving health outcomes.

      No clinical trial has evaluated the net benefit of testing versus no testing in improving health outcomes 

##  ***Links***

      * The National Comprehensive Cancer Network guidelines for CML [requires registration]: http://www.nccn.org/professionals/physician_gls/PDF/cml.pdf*

*The European Leukemia Network: http://www.leukemia-net.org/content/home/*

Last updated: 27^th^ October, 2010

## **Acknowledgments**

Please provide any relevant acknowledgments.

## **Funding information**

Supported through a contract with the Centers for Disease Control (for T. Trikalinos, T. Terasawa; author I. Dahabreh received no funding support).    

## **Competing interests**

The authors have declared that no competing interests exist.
